# Onset of common mental disorders and suicidal behavior following women’s first exposure to gender based violence: a retrospective, population-based study

**DOI:** 10.1186/s12888-014-0312-x

**Published:** 2014-11-18

**Authors:** Susan Rees, Zachary Steel, Mark Creamer, Maree Teesson, Richard Bryant, Alexander C McFarlane, Katherine L Mills, Tim Slade, Natacha Carragher, Meaghan O’Donnell, David Forbes, Derrick Silove

**Affiliations:** School of Psychiatry and Ingham Institute, University of New South Wales, Sydney, NSW 2170 Australia; Centre for Population Mental Health Research, Level 1 Mental Health Centre, Liverpool Hospital, South West Sydney Local Health District, Sydney, NSW 2170 Australia; St John of God Health Care, Richmond Hospital, North Richmond, NSW 2754 Australia; Department of Psychiatry, Australian Centre for Posttraumatic Mental Health, University of Melbourne, Melbourne, Australia; National Drug and Alcohol Research Centre, University of New South Wales, Sydney, Australia; Centre for Traumatic Stress Studies, University of Adelaide, Adelaide, Australia; School of Psychiatry and Ingham Institute, University of New South Wales, Sydney, NSW Australia

**Keywords:** Gender-based violence, Mental disorder, Temporal sequencing

## Abstract

**Background:**

Women exposed to gender-based violence (GBV) experience a high rate of common mental disorders and suicidal behaviour (“mental disturbance”). Little is known however about the timing of onset of mental disturbance following first exposure to GBV amongst women with no prior mental disorder.

**Methods:**

The analysis was undertaken on the Australian National Mental Health and Wellbeing Survey dataset (N = 8841). We assessed lifetime prevalence and first onset of common mental disorder and suicidal behaviour (mental disturbance) and exposure to GBV and its first occurrence based on the Composite International Diagnostic Interview Version 3 (WMH-CIDI 3.0). We used the Kaplan-Meier method to derive cumulative incident curves for first onset mental disturbance. The two derived subgroups were women who experienced GBV without prior mental disturbance; and women never exposed to GBV stratified to match the former group on age and socio-economic status.

**Results:**

For women with no prior mental disorder, the cumulative incidence curves showed a high incidence of all mental disturbances following first GBV, compared to women without exposure to GBV (all log rank tests <0.0001). Nearly two fifths (37%) of any lifetime mental disturbance had onset in the year following first GBV in women exposed to abuse. For these women, over half (57%) of cases of lifetime PTSD had onset in the same time interval. For GBV exposed women, half of all cases of mental disturbance (54%) and two thirds of cases of PTSD (66.9%) had onset in the five years following first abuse. In contrast, there was a low prevalence of onset of mental disturbance in the comparable imputed time to event period for women never exposed to GBV (for any mental disturbance, 1% in the first year, 12% in five years; for PTSD 3% in the first year, 7% in five years).

**Conclusions:**

Amongst women without prior mental disturbance, common mental disorders and suicidal behaviour have a high rate of onset in the one and five year intervals following exposure to GBV. There is a particularly high incidence of PTSD in the first year following GBV.

## Background

Women have high rates of common mental disorders and gender-based violence (GBV), public health problems that make a major contribution to the global burden of disease. These two health problems are closely inter-related, as indicated by a growing body of population-wide studies [1-3]. GBV refers to four types of related abuse including rape, other forms of sexual assault, intimate partner violence (IPV) and stalking [1,4-6]. Common mental disturbances associated with GBV include mood, anxiety and substance use disorders (SUDS) as well as suicidal behaviour [1-3].

As a severe form of trauma, GBV is an important precipitant of mental disturbance amongst women [2,7,8]. There is a gap in knowledge however concerning the temporal sequencing of first exposure to GBV and onset of mental disturbance [2,3]. In particular, clarification is needed about the timing of onset of mental disturbance following first exposure to GBV amongst the subpopulation of women with no prior mental health disturbances [2,3,7]. It seems likely but yet to be demonstrated that mental disturbances follow in close temporal proximity to initial GBV exposure [1,2,9]. If such a pattern can be confirmed, clinicians will be alerted to the need to monitor survivors of first GBV for onset of a range of mental disturbances in the early aftermath of the abuse. Given that first exposure to rape and sexual assault commonly occurs in childhood and adolescence, timely psychological intervention for mental disturbance following these abuses may prevent or mitigate risk of chronic mental disorder extending into later life.

Although longitudinal studies are the optimal approach for defining the chronological sequencing of GBV and mental disturbances, there are major ethical and methodological challenges in undertaking such investigations [10]. First exposure to GBV, particularly rape and other forms of sexual abuse, commonly occurs amongst young girls and adolescents within the context of the family of origin or the immediate social network. It clearly is not feasible to conduct door-to-door surveys making inquiry into these forms of abuse from potential survivors and members of their households [1,11]. Relying on the retrospective reports of adult women therefore represents the realistic alternative as long as the potential confound of recall bias is taken into account in interpreting the findings [12,13].

The second Australian Survey of Mental Health and Wellbeing (NSMHWB) (2007) offered an opportunity to undertake such a retrospective analysis [1]. Our focus is on common mental disorders (lifetime mood, anxiety, PTSD and substance use disorders) and suicidal behaviour (together, “mental disturbance”). We first distinguish women who experienced mental disturbances prior to GBV (group 1) from those who had not experienced any of the defined forms of mental disturbance prior to first abuse (group 2).

The aim of the study is to examine the trajectory of onset of mental disturbance in the latter group (group 2), with the remaining women (group 3) with no lifetime history of GBV providing a broad comparison group. We hypothesize that amongst women experiencing GBV for the first time (group 2), there will be a high incidence of first onset mental disturbance within one and five years following exposure.

## Methods

### Data collection

The source of the data was the second Australian National Survey of Mental Health and Well-being (NSMHWB), conducted in 2007 by the Australian Bureau of Statistics (ABS). The survey is an open access electronic database available for analysis and publication by appropriate academic institutions including the University of New South Wales. Following our formal application to the agency, the ABS granted us permission to access, analyse and publish the present data, issuing our team the Confidentialised Unit Record Files (CURF) to allow our use of the data [14,15].

The study involved a random, stratified, multistage area probability survey of persons aged 16 to 85 years drawn from the general Australian population [14,15]. Trained interviewers conducted face-to-face household interviews. One household member was selected for a computer assisted interview [15]. Of the 8,841 households surveyed, women comprised 4,551 respondents (a 65% response rate amongst women).

National legislation stipulates the ethical provisions governing ABS studies; the charter includes clear procedures for gaining permission from all participants and strict criteria for maintaining the privacy and safety of participants.

### Survey measure

The World Health Organisation Composite International Diagnostic Interview (WMH-CIDI 3.0) was used to assign lifetime diagnoses based on the Diagnostic and Statistical Manual IV (DSM-IV) [16]. Disorders are aggregated into the anxiety disorders (panic disorder, agoraphobia, social phobia, generalised anxiety disorder, obsessive-compulsive disorder and post-traumatic stress disorder), mood disorders (major depressive episode, dysthymia and bipolar affective disorder) and substance use disorders (harmful alcohol use, alcohol dependence and drug use disorders) [16].

DSM-IV aggregates PTSD with the other subtypes of anxiety under the rubric of the anxiety disorders. Given the trauma focus of our study, we deemed it important to undertake a sub-analysis to assess the specific impact of GBV on PTSD on its own. As PTSD requires a precipitating trauma to meet diagnostic criteria, and one of the GBVs assessed could be the index event, there is a theoretical risk that the inclusion of PTSD in the aggregated anxiety disorders might inflate associations between that category and GBV. We therefore undertook a preliminary analysis to examine whether inclusion or exclusion of PTSD from the aggregated anxiety disorder grouping influenced the outcome of our survival analyses. No substantive differences in the survival curves emerged when applying these alternative approaches. In our analysis of the anxiety disorders, we therefore included PTSD in order to allow comparison with the large number of epidemiological studies based on the CIDI that assess these disorders as a whole. In addition, we extracted persons with PTSD from the sample for an analysis of that disorder as a single category. A standard set of CIDI items inquired into suicidal behavior. In each instance, the timing of first onset of disorder or suicidal behavior was recorded.

Participants were asked to respond categorically (yes/no) to 29 potentially traumatic events (PTEs) they may have experienced over their lifetimes, a list that has been applied across countries participating in the World Mental Health Survey and that includes the four types of GBV [16]. Our previous analysis has shown that the four types of GBV are inter-correlated, that is, if a woman experienced one form, they were likely to be exposed to others over the course of their lives [1]. The question for intimate partner physical violence was: “Were you ever badly beaten up by a spouse or romantic partner?” Rape referred to sexual intercourse or penetration with a finger or object against the person’s will, or by use of threat or force, or when the person was too young to understand what was happening. The question for sexual assault referred to additional experiences of sexual abuse. Stalking was defined as being followed or kept track of in a manner that led to feelings of serious danger.

For each category of GBV, the first occurrence of the abuse was recorded. The number of subsequent exposures varied greatly across individual GBV types. Moreover, abuses such as intimate interpersonal violence and stalking often involve chronic patterns of behaviour, making it artificial to enumerate discrete episodes. For these reasons, we did not attempt to take into account the frequency of exposure to each type of GBV in our analyses.

### Statistical analyses

A full post-stratification weight for each record was computed by the ABS to adjust for the socio-demographic characteristics of the national population, the response rate, and the probability of being sampled [14]. For the purposes of the analysis, the whole sample of women was divided into (1) women who reported the onset of mental disturbance prior to exposure to GBV, a group not used in further analyses; (2) women exposed to GBV who did not have a prior mental disorder; and (3) the remainder of the sample who provided a comparison group of women not exposed to GBV. The latter group (group 3) was stratified to match women in group 2 according to age (13 × 5-year groupings) and socio-economic strata (5 categories). Socio-economic strata were based on a composite of variables comprising the Australian SEIFA index [17]. The procedure applied is analogous to a matched case control design except that stratification of the comparison group (group 3) was based on the full non-GBV exposed population, a procedure that allows maximization of sample size and as a consequence, more stable estimates than a single 1:1 matching. In cases where multiple women were exposed to GBV within a given age and socio-economic stratum in group 2, age of exposure was imputed proportionally to women in group 3.

Age of first occurrence of GBV for women in group 2 was imputed to non-GBV exposed women (group 3) according to the relevant matched age and socio-economic strata. Specifically, the time to event imputation procedure applied to group 3 was based on measurement of the period between first exposure to GBV and onset of each category of mental disturbance for women in Group 2. To be consistent with group 2, age at study interview was applied as the censored time to event for women in the comparison group (group 3) who did not develop a mental disorder.

For groups 2 and 3, we assessed trajectories of onset of mental disturbances by computing cumulative incident curves according to the Kaplan-Meier method for calculating survival curves, an approach that is consistent with other studies in the literature examining cross-sectional data of this type [18]. The log rank statistic was used to test for statistical differences in curves for any mental disturbance and its subcomponents (depression, anxiety, etc.) across the two samples. Normalised post-stratification weighting was used for all statistical modelling [19]. Analyses were carried out using SAS V9.3 [20].

## Results

Of the whole sample of 8841 participants, 4451 were women (overall response 60%, 65% for women). The socio-demographic characteristics of the whole sample are displayed in Table 1. Over a quarter of women 1218 (27.4%) reported at least one lifetime exposure to GBV [1]. The lifetime prevalence of GBV was highest (35.8%) for women aged 30 to 49 years and lowest (14.5%) for women aged 65 years or older. In relation to marital status, married women had the lowest prevalence of GBV. GBV was more prevalent amongst the most socio-economically disadvantaged women.

**Table 1:** **Social-demographic characteristics of sample**

	***n = 4451*** **(100%)**
Age group	
65 +	699 (15.7)
50-64	1082 (24.3)
30-49	1629 (36.6)
16-29	1042 (23.4)
Marital status	
Never married	1297 (29.1)
Others	809 (18.2)
Married	2345 (52.7)
Immigration status	
Australian born	3250 (73.0)
Immigrant from English speaking country	450 (10.1)
Immigrant from Non-English speaking country	751 (16.9)
Highest level of post-school educational attainment	
University degree or higher	893 (20.1)
Diploma	599 (13.4)
Vocational	805 (18.1)
None	2154 (48.4)
Labour force full-time/part time	
Employed	2650 (59.5)
Unemployed	98 (2.2)
Not in the labour force	1704 (38.3)
Index of Disadvantage	
1st quintile	757 (17.0)
2nd quintile	833 (18.7)
3rd quintile	883 (19.8)
4th quintile	927 (20.8)
5th quintile	1050 (23.6)

Table 2 shows the prevalence of mental disturbance amongst the three groups of women. Group 1 (those who reported onset of any mental disturbance prior to GBV exposure) comprised 310 women. Group 2, women exposed to GBV who did not have prior mental disturbance, comprised 929 participants; of these, 486 (52.3%) experienced a lifetime mental disturbance. Group 3, the comparison group of women not exposed to GBV, comprised 3213 women, with a lifetime prevalence of mental disturbance of 885 (27.5%).

**Table 2:** **Prevalence of mental disturbances for the three derived subpopulations** ^†^

	**Group 1**		**Group 2**		**Group 3**	
	**n**	**%**	**n**	**%**	**n**	**%**
Number	310		929		3213	
Any	310	100	486	52.3	885	27.5
Mood	102	32.9	311	33.5	401	12.4
Anxiety	222	71.6	351	37.8	524	16.3
PTSD	74	23.8	232	25.0	138	4.2
SUD	79	25.4	267	28.7	273	8.4
Suicide	60	19.3	82	8.8	24	0.7

G1 - Women who reported the onset of mental disturbance prior to exposure to GBV.

G2 - Women exposed to GBV who did not have prior mental disorder.

G3 - Comparison group of women not exposed to GBV.

^†^Percentages refer to the number of persons in the group with the relevant mental disturbance as a proportion of the total number in that group.

**Figure 1:**
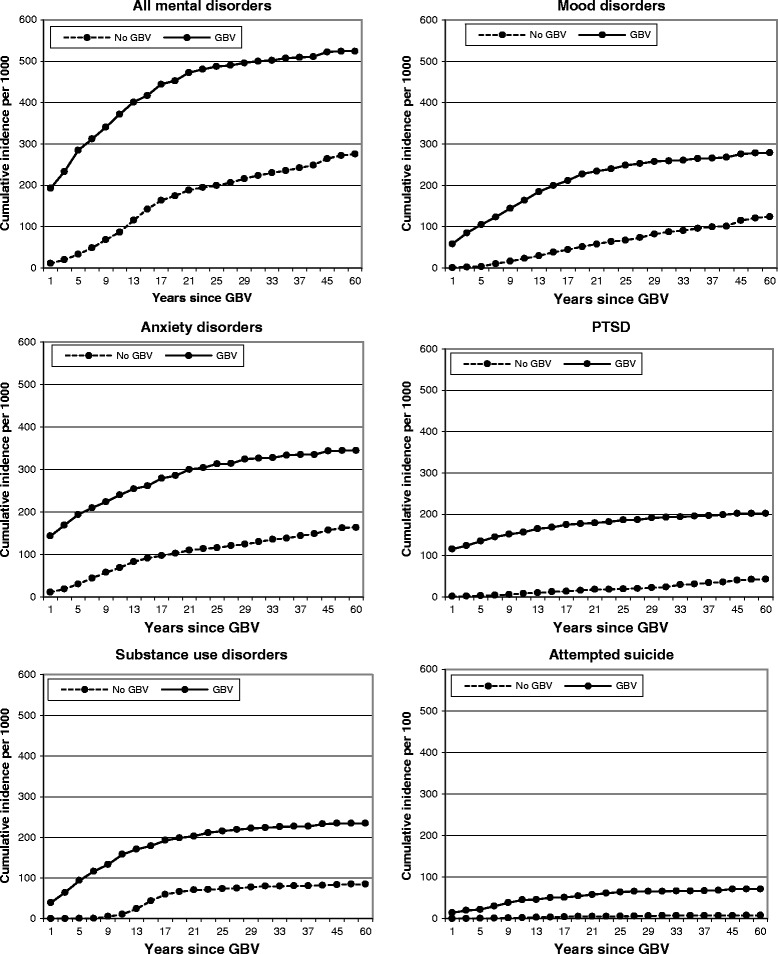
**Survival Curves comparing women exposed and not exposed to GBV with mental disorders.** Survival Curves showing cumulative incidents (‘000) (Y axis) for women exposed to GBV without previous mental disorder (n=929), compared to women with no exposure to GBV (n=3213).

All further analyses involved groups 2 and 3 only. Figure 1 displays the graphs for the cumulative incident curves for the GBV-exposed women without prior mental disturbance (group 2) and women not exposed to GBV (group 3). There were statistical differences for all trajectories (for combined and individual indices of mental disturbance) in comparisons between groups 2 and 3 (all log rank tests p < 0.0001). The key differences noted across groups were: the overall lifetime rates of all forms of mental disturbance were much higher for GBV exposed women (group 2) than the non-exposed women (group 3); in group 2, for most forms of mental disturbance, the gradient of onset was steepest in the early years following first exposure to GBV with the curves levelling out thereafter, approximating the slope of group 3; the exception was suicidal behaviour where the incidence for group 2 remained high for a prolonged period following first abuse.

Table 3 records onset of mental disturbances for the time intervals of one year and five years following first exposure to GBV for group 2 and from the imputed time of event for group 3. In the first year following initial GBV, the rates for group 2 were: any mental disorder, 179 (37% of lifetime prevalence for that group), mood disorder 65 (20.9%), anxiety disorder, 146 (33%), PTSD, 135 (57%), SUDs, 44 (16%), and suicidal behaviour, 16 (19.5%). The cumulative five year incidence following first GBV in group 2 was 264 (54%) for any disorder, 117 (27.6%) for mood disorder, 197 (56%) for anxiety disorder, 158 (66.9%) for PTSD, 107 (40%) for SUDs, and 25 (30.4%) for suicidal behaviour.

**Table 3:** **Incidence of mental disturbance in one and five year time intervals after event for women experiencing first GBV (n = 929) and those with no exposure to GBV**

**Women with first GBV and no prior mental disturbance (n = 929)**												
	**All MD**		**Mood**		**ANX**		**PTSD**		**SUDs**		**Suicide**	
	**(n)**	**%LT**	**(n)**	**%LT**	**(n)**	**%LT**	**(n)**	**%LT**	**(n)**	**%LT**	**(n)**	**%LT**
1 year	179	36.8%	65	20.9%	146	33.0%	135	57.2%	44	16.4%	16	19.5%
5 year	264	54.3%	117	37.6%	197	56.1%	158	66.9%	107	40.0%	25	30.4%
Lifetime	486		311		351		232		267		82	
**Women not exposed to GBV (n = 3213)**												
	**All MD**		**Mood**		**ANX**		**PTSD**		**SUDs**		**Suicide**	
	**(n)**	**%LT**	**(n)**	**%LT**	**(n)**	**%LT**	**(n)**	**%LT**	**(n)**	**%LT**	**(n)**	**%LT**
1 year	36	4%	3	0.7%	35	6.6%	4	2.9%	0	0.0%	0	0.0%
5 year	106	12%	12	3.0%	97	18.5%	9	6.5%	1	0.3%	1	4.1%
Lifetime	885		401		524		138		273		24	

For the comparison group (group 3), rate of onset of any disorder during the imputed time period was 36 (1.1% of lifetime disorder in that group) for one year, and 106 (12.0%) within five years; for mood disorder 3 (0.7%) within one year, and 12 (3.0%) within 5 years; for anxiety disorders, 35 (6.6%) for one year, and 97 (18.5%) for five years; and for PTSD 4 (3%) for one year, and 9 (6.5%) for five years. For substance use disorders, no women in group 3 had onset within one year, and 1 (0.3%) within five years. In this group, there were no suicidal acts within the first year, and one (4%) in the five year interval.

## Discussion

Our findings indicate that amongst the two thirds of GBV-exposed women who have not experienced one of the designated mental disturbances prior to the abuse, a substantial portion manifested at least one of these problems within one and five years following initial exposure to abuse. Notably, for PTSD, the majority of lifetime cases (57%) had onset in the year following first exposure to GBV, an important finding given the specific relationship of trauma to that diagnostic category [21,22]. In addition to common mental disorders (depression and anxiety), first onset GBV was associated with the proximate onset of alcohol and drug use problems amongst the sample of women [23,24]. The initially steep gradient of the cumulative incidence curves following GBV levelled off over time, gradually approximating that of women not exposed to GBV, suggesting that the first experience of GBV may have been a salient factor in the pattern of onset of mental disturbance. Women exposed to first GBV showed a high rate of onset of self-harming behaviour, in this instance, extending over a protracted period following the first experience of abuse.

The strengths and limitations of the study need to be considered. The study was based on a large, nationally representative sample of women. The response rate of 65% is comparable to other international mental health surveys of this kind [15]. The study excluded the homeless, those residing in institutions, and the severely mentally ill [15]. Populations living in remote and rural areas were under-represented. The study was undertaken in a high income, English-speaking country, making it important to extend inquiry to low and middle income countries in order to establish significance of the findings in those settings.

The CIDI-3 has been used in multiple national surveys worldwide and diagnoses have been validated against gold standard structured clinical interviews [25]. Identification of individual forms of GBV was based on single items rather than on comprehensive interview used in international studies [6,26]. Nevertheless, the rates of GBV recorded are similar to those obtained in other Australian surveys of violence using more elaborate measures to detect these abuses [27]. The items relating to IPV were restricted in the survey to severe physical abuse and did not include psychological abuse, a component of IPV [28,29]. Stalking, a form of psychological abuse, was included in our analysis as a type of GBV [5]. The study focused on the sequence and timing of a mental disorder following exposure to a GBV and therefore did not investigate the factors responsible for mental disorder in those where disturbances occurred prior to GBV exposure, further research is needed to identify the characteristics of that subpopulation [2,3,10,30].

Our analyses were based on the retrospective recall of participating women. While it remains possible that women with a range of mental disorders are more likely to recall adverse events in their earlier lives, there is evidence that memory for the more severe forms of trauma such as GBV is not subject to such bias [31,32]. It is theoretically possible that women were motivated by a retrospective “search for meaning” to link the timing of onset of mental health problems to the first occurrence of GBV. It should be noted however, that with the exception of PTSD, the data relating to GBV were elicited within a general inventory of trauma events assessed in a specific of an extensive interview. Importantly, the trauma events section was disconnected in sequence from that evaluating mental disorder and suicidal behaviour. It would be expected therefore that the chronology of the interview would reduce the likelihood of participants making intentional links between the timing of first GBV and the onset of mental disturbances.

The cross-sectional nature of the study cautions against drawing a direct causal link between the first occurrence of GBV and the timing of onset of the mental disturbances measured. In addition, the measure used did not include indices of wider forms of childhood adversity that could account in part for both exposure to GBV and risk of mental disorder. Moreover, our analyses did not include the possible impact of other traumas and stresses occurring in later life [33]. A conservative interpretation of our findings therefore is that first exposure to GBV represents a marker or sentinel event that signals a high risk of onset of a range of common mental disturbances in the early years following the abuse. Further research will be needed to map out more precisely the pathways and intervening factors that influence these outcomes.

Caveats notwithstanding, our findings provide important new data that offer guidance to researchers and practitioners in the field. The two thirds of women exposed to GBV prior to experiencing one of the designated mental disturbances had a high risk of onset of one or more of these problems in the early aftermath of the abuse, that is, within a one year and five year time interval. It is common for rape and sexual assault to occur for the first time in childhood and adolescence, whereas IPV and stalking often have their onset in early adulthood [1]. Our findings therefore underline the imperative for primary health care providers to monitor girls, adolescents and young women after first exposure to GBV in order to ensure the timely detection of incipient mental disorders and suicidal behaviour in the early years following the abuse. Similarly, the identification of first onset common mental disturbance in girls, adolescents and young women should alert practitioners to the possibility of GBV being a key and possibly undisclosed element of the recent history, requiring sensitive assessment to ensure detection.

We note however that although a range of strategies have been trialled with the aim of mitigating the social, economic and safety factors related to GBV, evidence for the for the efficacy of these interventions remains scant. Lack of specific attention to the mental health aspects of the problem may be partially responsible for poor overall outcomes [10,34-38]. Our findings therefore support the need to devise and test novel programmes that match early detection of GBV with timely multidisciplinary interventions that address the inter-related sequelae including risk of further abuse, adverse social consequences, and possible mental disturbances, with a particular focus on the phases of childhood, adolescence and early adulthood. In order to achieve better outcomes, specialist agencies for GBV and general mental health services need to work in closer liaison to ensure that a comprehensive approach is pursued to addressing the multifaceted nature of this problem.

## Conclusions

It is well-established that women have much higher rates of exposure to GBV than men.

Our findings are novel in showing a close temporal relationship between first exposure to GBV and onset of a wide range of mental disturbances amongst women. The results support the need for developing policy and innovative strategies aimed at addressing the dual challenge of responding to GBV and the associated mental health consequences for women in a comprehensive and integrated manner [8,39]. At a population level, focusing on preventing GBV amongst women may assist in averting onset of a range of common mental disturbances that contribute substantially to the global burden of disease [40,41].

## Abbreviations

GBV: Gender-based violence
SUDs: Substance use disorders
PTSD: Post-traumatic stress disorder
